# Extrinsic innervation of the pelvic organs in the lesser pelvis of human embryos

**DOI:** 10.1111/joa.13229

**Published:** 2020-06-27

**Authors:** Nutmethee Kruepunga, Jill P. J. M. Hikspoors, Cindy J. M. Hülsman, Greet M. C. Mommen, S. Eleonore Köhler, Wouter H. Lamers

**Affiliations:** ^1^ Department of Anatomy and Embryology Maastricht University Maastricht The Netherlands; ^2^ Department of Anatomy Faculty of Science Mahidol University Bangkok Thailand; ^3^ Tytgat Institute for Liver and Intestinal Research Academic Medical Centre Amsterdam The Netherlands

**Keywords:** hindgut, hypogastric plexus, median sacral artery, neural crest cells, pelvic pouch, splanchnic nerves, umbilical arteries

## Abstract

Realistic models to understand the developmental appearance of the pelvic nervous system in mammals are scarce. We visualized the development of the inferior hypogastric plexus and its preganglionic connections in human embryos at 4–8 weeks post‐fertilization, using Amira 3D reconstruction and Cinema 4D‐remodelling software. We defined the embryonic lesser pelvis as the pelvic area caudal to both umbilical arteries and containing the hindgut. Neural crest cells (NCCs) appeared dorsolateral to the median sacral artery near vertebra S1 at ~5 weeks and had extended to vertebra S5 1 day later. Once para‐arterial, NCCs either formed sympathetic ganglia or continued to migrate ventrally to the pre‐arterial region, where they formed large bilateral inferior hypogastric ganglionic cell clusters (IHGCs). Unlike more cranial pre‐aortic plexuses, both IHGCs did not merge because the 'pelvic pouch', a temporary caudal extension of the peritoneal cavity, interposed. Although NCCs in the sacral area started to migrate later, they reached their pre‐arterial position simultaneously with the NCCs in the thoracolumbar regions. Accordingly, the superior hypogastric nerve, a caudal extension of the lumbar splanchnic nerves along the superior rectal artery, contacted the IHGCs only 1 day later than the lumbar splanchnic nerves contacted the inferior mesenteric ganglion. The superior hypogastric nerve subsequently splits to become the superior hypogastric plexus. The IHGCs had two additional sources of preganglionic innervation, of which the pelvic splanchnic nerves arrived at ~6.5 weeks and the sacral splanchnic nerves only at ~8 weeks. After all preganglionic connections had formed, separate parts of the inferior hypogastric plexus formed at the bladder neck and distal hindgut.

## Graphical abstract

The umbilical arteries separate the lesser pelvis from the abdomen. Development of the inferior hypogastric plexus is divided into migration of neural crest cells (blue dots) to para‐ and pre‐arterial positions, followed by association of the ganglionic cell cluster with the superior hypogastric nerve, then the pelvic splanchnic nerves, and finally the sacral splanchnic nerves. Only thereafter nerves and ganglionic cells extend from this plexus along the bladder wall and the caudal hindgut.

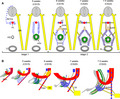

## 1 INTRODUCTION

The pelvic organs, which occupy the lesser or 'true' pelvis, are innervated by the autonomic nervous system. The caudal portion of the vagal neural crest cells is the major source of neural crest‐derived cells (NCCs) of the intrinsic enteric nervous system (ENS; Durbec *et al*. 1996; Anderson *et al*. 2006; Simkin *et al*. 2013; Espinosa‐Medina *et al*. 2017). However, studies in chicken (Le Douarin and Teillet, 1973; Burns and Le Douarin, 1998), and subsequently in rodents (Serbedzija *et al*. 1991; Anderson *et al*. 2006), have shown that NCCs that originate distal to vertebral level L1‐2, which corresponds to somite 28 in chicken (Le Douarin and Teillet, 1973) and somite 24 in mice (Dong *et al*. 2006), contribute to the mature ENS of the colon. The timeline of the development and distribution of these sacral NCCs in mammals is described in most detail for rodents. In mice, these cells emigrate from the neural tube at embryonic day (ED) 9.0–9.5, aggregate in the para‐aortic region at ED10.5–11.0, and form the pelvic ganglia ventrolateral to the hindgut at ED11.5–12.5. From here they enter the wall of the gut or base of bladder at ED13.5–14.0 and have colonized the entire postcoecal gut at ED14–14.5. Soon thereafter, differentiation into intramural ganglia and the formation of smooth muscle layers begins (Serbedzija *et al*.^1991^; Kapur, 2000; Dong *et al*. 2006; Wang *et al*. 2011; Erickson *et al*. 2012). Vagal NCCs emigrate from the neural tube at ED8.0–8.5, enter the gut at ED9.5, arrive at the coecum at ED10.5, bypass the coecum via the dorsal mesentery to move into the postcoecal gut at ED12.5, reach the midpoint of the colon by ED13.5 and the hindgut at ED14.5 (Kapur *et al*. 1992; Durbec *et al*. 1996; Young *et al*. 1998; McKeown *et al*. 2001; Druckenbrod and Epstein, 2005; Nishiyama *et al*. 2012). These data imply that the sacral NCCs take two to three times longer to move from the neural tube to the wall of the gut, but migrate approximately twofold faster through the postcoecal gut than the vagal NCCs.

A number of studies address sacral NCC migration (Kuntz, 1952; Kimmel and McCrea, 1958; Okamoto and Ueda, 1967; Arango‐Toro and Domenech‐Mateu, 1993) and pelvic nerve development (Browne, 1953; Pearson and Sauter, 1970; Arango‐Toro and Domenech‐Mateu, 1993; Kinugasa *et al*. 2008) in human embryos. From these studies, the general picture emerges that the development of the pelvic nervous system in humans is similar to that in rodents. Unfortunately, relatively few embryos were studied and the staging was rather imprecise. Perhaps even more limiting is the fact that these studies did not provide spatial models [apart from a single, elegant wax model of an 8‐week‐old embryo in Arango‐Toro and Domenech‐Mateu (1993)]. It is, therefore, difficult to appreciate topographic relations between the gut, nerves and pelvic organs.

In the present study, we investigated the development of the inferior hypogastric plexus and its preganglionic connections with the central nervous system: the hypogastric, pelvic and sacral splanchnic nerves. Recently, the pelvic splanchnic nerves were shown to be developmentally and phenotypically sympathetic (Espinosa‐Medina *et al*. 2016), defining all three inputs as sympathetic. For this reason, we carefully mapped the timeline of their appearance and contact with the bilateral pelvic ganglionic cell clusters. Another reason to map the development of the hypogastric plexus and nerves was the difference in the timelines of the vagal (cranial) and sacral (caudal) contributions to colonic innervation. We further defined the topographical boundaries of the lesser pelvis in the embryo.

## 2 MATERIALS AND METHODS

### 2.1 Embryos

This study was undertaken in accordance with the Dutch regulations for the proper use of human tissue for medical research purposes. Well‐preserved anonymous human embryos and foetuses, donated for scientific research, from the historical collections of the Departments of Anatomy and Embryology, Leiden University Medical Centre, the Amsterdam University Medical Centres, location Academic Medical Centre and Radboud University, Nijmegen, The Netherlands, and from the University of Göttingen, Germany (Blechschmidt Collection; https://doi.org/10.3249/ugoe‐publ‐2) were studied (Table 1). In addition, digital images of carefully staged human embryos from the Carnegie collection (Washington, DC, USA) were included from the Digitally Reproduced Embryonic Morphology project (http://virtualhumanembryo.lsuhsc.edu).

**Table 1:** Metadata of human embryos and foetuses that were studied

Stage	Days	Embryo	Fixation	Staining	Plane	Source
CS10	28	S6330	Formalin	Ehrlich's H	Transv	DREM
CS11	29	S6344	Formalin	CA	Transv	DREM
CS12	30	S8943	Zenker's fix	H & E	Transv	DREM
CS13	32	S836	HgCl_2_	CA	Transv	DREM
CS14‐early	33	S2201	Formalin	H & A	Transv	AMC
CS14‐mid	34	S5029	Formalin	H & A	Sagittal	AMC
CS14‐mid	34	S168	Bouin's fix	H & E	Transv	LUMC
CS14‐mid	34	1950‐09‐13		H & E	Sagittal	Göttingen
CS14‐late	35	1958‐12‐22		H & E	Sagittal	Göttingen
CS14‐late	35	1961‐06‐13		H & E	Transv	Göttingen
CS14‐late	35	S6502	Souza's fix	H & E (or +Ag)	Transv	DREM
CS15‐early	36	S721	Zenker's fix	H & E (or +Ag)	Transv	DREM
CS15‐early	36	S79	Formalin	H & E	Transv	LUMC
CS15‐early	36	1945‐10‐26		H & E	Transv	Göttingen
CS15‐early	36	1957‐10‐31		H & E	Transv	Göttingen
CS15‐late	37	S2213	Formalin	H & A	Transv	AMC
CS16	39	S5032	Formalin	H & A	Sagittal	AMC
CS16	39	S6517	Corrosive CH_3_COOH	CA	Transv	DREM
CS16	39	S39	Formalin	H & E	Transv	LUMC
CS17	41	S6520	Corrosive CH_3_COOH	CA (or +Ag)	Transv	DREM
CS18	44	S97	Bouin's fix	H & E	Transv	LUMC
CS18	44	S4430	Corrosive CH_3_COOH	CA	Transv	DREM
CS19	46	S9325	Acetic formalin	Azan & Ag	Transv	DREM
CS20	49	S2025	Bouin's fix	H & A	Transv	AMC
CS20	49	S462	Formalin	CA	Transv	DREM
CS20	49	S34	Formalin & Bouin's fix	H & E	Sagittal	LUMC
CS21	51	S4090	Formalin	CA	Transv	DREM
CS22	53	S48	Formalin	H & E	Transv	LUMC
CS22	54	S983	Formalin	H & E	Transv	DREM
CS23	56	S4141	Formalin	H & A	Transv	AMC
CS23	56	S9226	Formalin	Azan	Transv	DREM
CS23	56	S88	Formalin & Bouin's fix	H or PAS or Azan	Sagittal	Radboud Medical Centre

AC, alum cochineal (i.e. carmine); AMC, Academic Medical Centre; CS, Carnegie stage; DREM, Carnegie collection from the Digitally Reproduced Embryonic Morphology project; Göttingen, Department of Anatomy and Embryology, Göttingen; H & A, haematoxylin and azophloxine; H & E, haematoxylin and eosin; LUMC, Leiden University Medical Centre; PAS, Periodic acid–Schiff stain.

The estimated post‐fertilization ages of the embryos are based on (O'Rahilly and Muller, 2010). The additions 'early', 'mid' and 'late' are meant to indicate that, within these stages, the development of the gut and enteric nervous system of 'late' embryos was more advanced than that of 'early' embryos. The corresponding age was chosen from the range of developmental days attributed to that stage (O'Rahilly and Muller, 2010). CS14 in particular is noted for its remarkable number of developmental events.

### 2.2 Image acquisition, 3D reconstruction and visualization

Human embryos of between 4 and 8 weeks post‐fertilization development were investigated. The modified O’Rahilly's criteria were used to define the Carnegie stage (CS) of development and post‐fertilization age (O'Rahilly and Muller, 2010; Table 1). A graph relating the CSs of human embryos to days of development in mice or Hamilton–Hamburger stages (Hamburger and Hamilton, 1951) in chicken are found in Figure S1. Serial sections from the named historical collections were digitized with an Olympus BX51 or BX61 microscope and the Dotslide program (Olympus) to provide high‐resolution digital images. Serial sections of the Blechschmidt collection were digitized with a Zeiss Axio Scan.Z1 (Carl Zeiss Microscopy). All digital images were transformed into greyscale ‘JPEG’ format and imported into Amira3D (version 6.5; FEI Visualization Sciences Group Europe). The imported images were aligned automatically with the least‐squares function and then manually optimized by correction for the embryonic curvature with the aid of photographs and magnetic resonance images of the same stages of human embryos (Pooh *et al*. 2011). Structures of interest were segmented manually and used to generate 3D shapes with the Amira3D program. To eliminate the distracting noise in the Amira3D output attributable to section processing and stacking, Amira3D polygon meshes were exported via ‘vrml export’ to Cinema 4D (version R21; MAXON Computer GmbH) and remodelled using the Amira3D model as a template. Concurrent visualization of the Amira3D template and the remodelled Cinema4D model in Cinema 4D was used to verify the accuracy of the Cinema4D models (Figure S2). The Cinema4D models were transferred via ‘wrl export’ to Adobe Acrobat version 9 (http://www.adobe.com) to generate interactive 3D PDF files, which are a user‐friendly format for 3D visualization (Figures S3 and S4). We mostly refer in the text to the figures to relate histology to developing structures, but encourage the reader to simultaneously inspect the interactive PDFs, because their rotational options ('live' images) allow a much better understanding of the complex local topography than the 'still' pictures in the images. Note that the orientation of the 3D models is aligned according to the (vertical) axis through segments (vertebrae) T1 and L1.

### 2.3 Terminology

Intestinal development in avian (Southwell, 2006) and mammalian embryos (Soffers *et al*. 2015) proceeds in a similar fashion, with the midgut or primary loop extending into the coelomic cavity of the umbilical cord as the so‐called 'umbilical hernia'. The main differences appear to be the formation in birds of only a single (duodeno‐jejunal) secondary loop and two coecal diverticula, whereas four secondary gut loops and a single coecal diverticulum form in mammals. In both avian and mammalian embryos the cells of the vagal neural crest colonize both 'pre‐umbilical' and 'post‐umbilical' parts of the gut, with the position of the vitelline duct forming the landmark between 'pre‐' and 'post‐'. The sacral NCCs arise distal to vertebral level L1‐2 in both birds and mammals (Le Douarin and Teillet, 1973; Dong *et al*. 2006) and only colonize the 'post‐umbilical' gut in a caudocranial gradient that becomes progressively steep during development (Le Douarin and Teillet, 1973; Burns and Le Douarin, 1998; Anderson *et al*. 2006). Whereas the avian vitelline duct remains present and patent until hatching (Esteban *et al*. 1991), the mammalian vitelline duct already disappears at ~CS15 in rodent (Lamers *et al*. 1987) and human (Soffers *et al*. 2015) embryos. Due to the disappearance of the vitelline duct in mammalian embryos, the cranial boundary of the post‐umbilical gut can no longer be delineated accurately. Accordingly, the part of the gut colonized by sacral NCCs is variously referred to as the hindgut (Young and Newgreen, 2001; Wang *et al*. 2011), post‐coecal hindgut (McKeown *et al*. 2001), or colorectum (Young and Newgreen, 2001). In agreement with our accompanying study, we will identify the mammalian equivalent of the avian post‐umbilical gut as the distal loop of the midgut and hindgut.

Some terminology is confusing because embryonic and definitive structures differ markedly in appearance. Relevant for the present study are the superior hypogastric nerve and plexus, the inferior hypogastric (ganglionic) cluster and plexus, and the hindgut and rectum. The superior hypogastric plexus acquires its definitive configuration upon the division of the single large splanchnic nerve into many smaller nerve strands during CS18–20. Similarly, we describe the inferior hypogastric plexus as a cluster of ganglionic cells until it becomes populated by nerves at CS20.

Our detailed study of the extrinsic innervation of the caudal part of the gut showed that the junction of the mid‐ and hindgut corresponded with the location of the stem of the inferior mesenteric artery (IMA) rather than the cranial end of its left colic branch. The stem of the IMA branches from the aorta at vertebral level T12‐L1 during CS15 and has descended to L2‐3 at CS20 (Evans, 1912). This level corresponds, in turn, with the cranial boundary of the sacral neural crest (Le Douarin and Teillet, 1973; Dong *et al*. 2006) and, as we will show, with the rectosigmoidal junction rather than the transverse colon in the adult. We will describe the development of the junction between the mid‐ and hindgut trunk in more detail in a separate study.

## 3 RESULTS

In Kruepunga *et al*., in press), we identified the neural crest‐derived ganglionic cells in the thoraco‐lumbar region by their topography and intense staining properties. In this study, we investigated the appearance and migration of NCCs in the caudal‐most portion of the body, the lesser pelvis.

### 3.1 Boundaries of the lesser pelvis in the embryo

**FIGURE 1:**
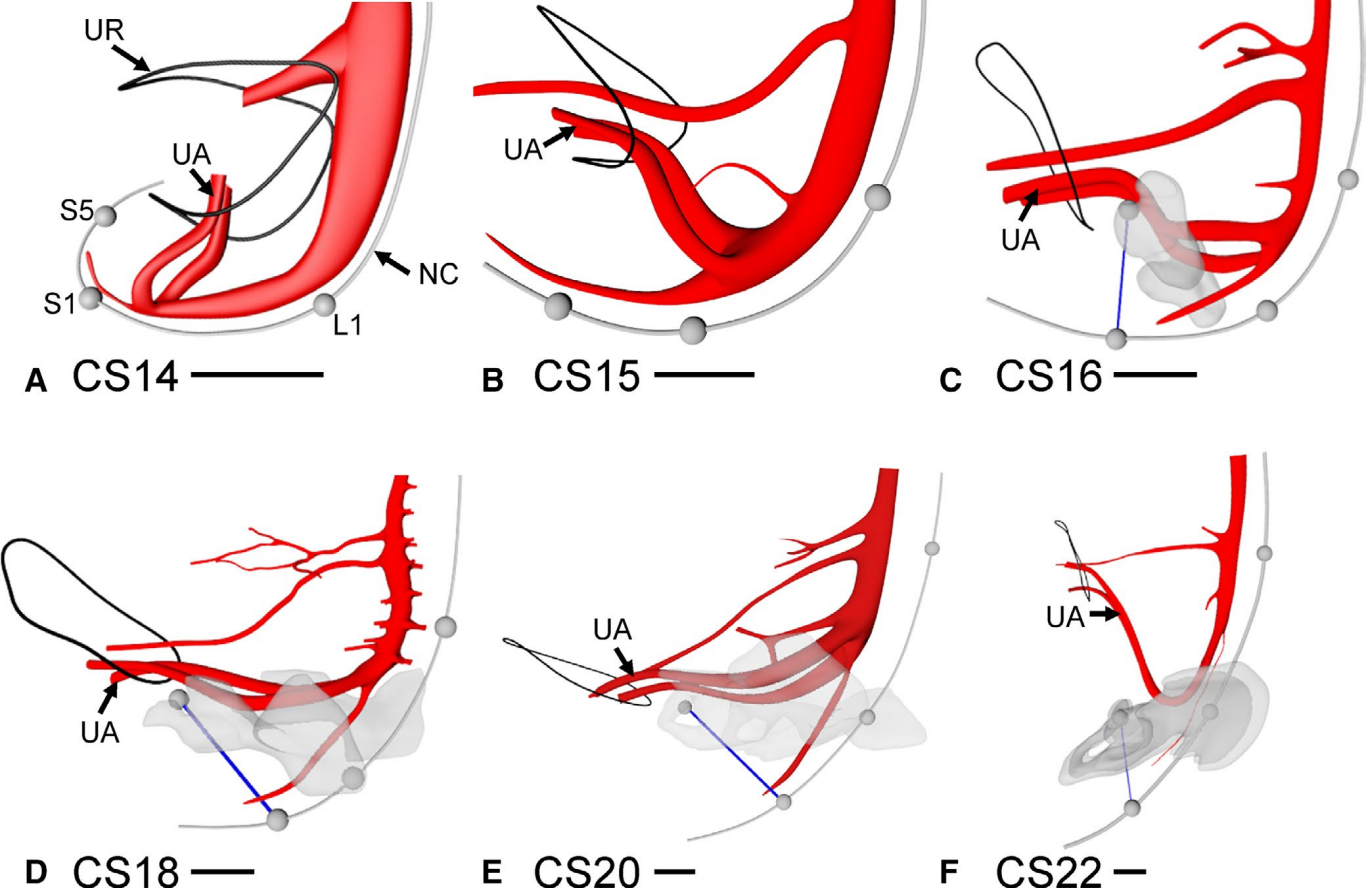
The course of the umbilical arteries defines the upper boundary of the lesser pelvis. Panels a–f show side views of the dorsal aorta and its major ventral branches between Carnegie Stage (CS)14 and CS22, with the notochord representing the embryonic curvature and the grey spheres marking segments L1, S1 and S5. At CS14 (33 days; panel a) the plane through the bifurcation of the umbilical arteries was orthogonal to a frontal plane through the aorta. As the caudal body axis unfolded (Kruepunga *et al*. 2018), the angle between both planes straightened to 160–170° at CS22. Concomitantly with the unfolding of the embryonic axis, the line through the subpubic arch and vertebra S5 unfolded from CS16 onwards (panels c–f). Note that the unfolding process plateaus in the lumbar region at CS17, but continues in the sacral region. Bars = 500 µm. NC, notochord; UA, umbilical arteries; UR, umbilical ring. [Colour figure can be viewed at wileyonlinelibrary.com]

We define the lesser pelvis in the embryo as the pelvic area caudal to both umbilical arteries (Figure 1). The umbilical arteries branched away from the dorsal aorta at the level of lumbar vertebra 4 (L4), irrespective of the developmental stage of the embryo. On their course to the umbilical cord, the umbilical arteries passed the insertion of the Wolffian ducts into the urogenital sinus and the allantois laterally (Figures S2 and S3). Until CS15‐late (~37 days of development), the plane through both umbilical arteries ('UA' in Figure 1) was almost perpendicular to the frontal plane through the dorsal aorta at the bifurcation. Between CS16 and CS23 (39–56 days of development), the angle between both planes gradually disappeared, concomitant with the unfolding of the caudal part of the body axis. The caudal unfolding involved the lumbar and sacral regions until CS17 and was confined to the sacral region between CS17 and 10 weeks of development (Kruepunga *et al*. 2018). A striking feature of the umbilical arteries was the absence of extrinsic enteric nerves on their ventral side (not shown). Up to and including CS15 (~37 days of development), the umbilical arteries, therefore, also impressed as a barrier between the abdominal and pelvic nerves, but concomitant with the local unfolding of the body axis, the bifurcation was crossed ventrally by the developing hypogastric nerve.

Mesenchymal cells caudolateral to the umbilical arteries started to condense to form the cartilaginous template of the hip bones at CS16 (Figure 1). The position of the hip bones also followed the unfolding process up to CS20, as can be deduced from the change in position of the pubococcygeal line through the pubic arch and vertebra S5 (blue lines in Figure 1). At 7.5 weeks (CS22), the proximal portion of the umbilical arteries, therefore, occupied the position of the common iliac arteries (Figure 1), which defines the upper boundary of the lesser pelvis in the adult. Of relevance for their landmark function, the umbilical arteries produced caudolateral, but no cranioventral branches. The marked curvature of the umbilical arteries in the sagittal plane at CS22, with the most caudal point lateral to the bladder trigone [the most cranial region of the urogenital part of the cloaca (Kruepunga *et al*. 2018)] showed that the straightening of the body axis in the late embryonic period represented axial growth of the dorsal part of the body, which was matched ventrally by the development of the ventral body wall from the lower thoracic dermomyotomes (Mekonen *et al*. 2015) and the fundus of the bladder from the allantois (van der Putte, 2005; Kruepunga *et al*. 2018). We observed the configuration of the umbilical arteries, as shown in panel E in another CS22 embryo, while the configuration as shown in panel F was seen all four CS23 embryos studied, showing that the formation of the infra‐umbilical ventral body wall proceeds rapidly.

We reported earlier that one of the features that accompanied early herniation of the midgut (CS14 and CS15) was a thinning of the dorsal mesentery between the superior and inferior mesenteric arteries (Soffers *et al*. 2015; Hikspoors *et al*. 2019). The thin base of the midgut mesentery and the thick base of the hindgut mesentery are well visible in Figure S5 (asterisks in panels B and C). This feature persisted for the next 3 weeks (Figures 3E and 4F–H) and demarcated the transition of the herniated colon and the non‐herniating hindgut (CS16–20). At CS20, when the IMA had branched into the left colic and superior rectal arteries, the stem of the IMA pointed to the transition of herniated and non‐herniated parts of the caudal gut (Figure S3B). At CS23, a leftward‐oriented colic loop with a thin mesentery started to form ventral to the left gonad. Caudally, the return of this loop to the midline co‐located with the thickening and shortening of the dorsal mesentery, with the plane through both umbilical arteries described in the previous paragraph, and with the appearance of the superior hypogastric nerve at the intestinal base of the dorsal mesentery that will be described in the paragraph on the 'Formation of the nerve fibre network of the inferior hypogastric plexus'. These three features characterize the plane that represents the superior boundary of the lesser pelvis.

### 3.2 Formation of the inferior hypogastric ganglionic‐cell cluster

**FIGURE 2:**
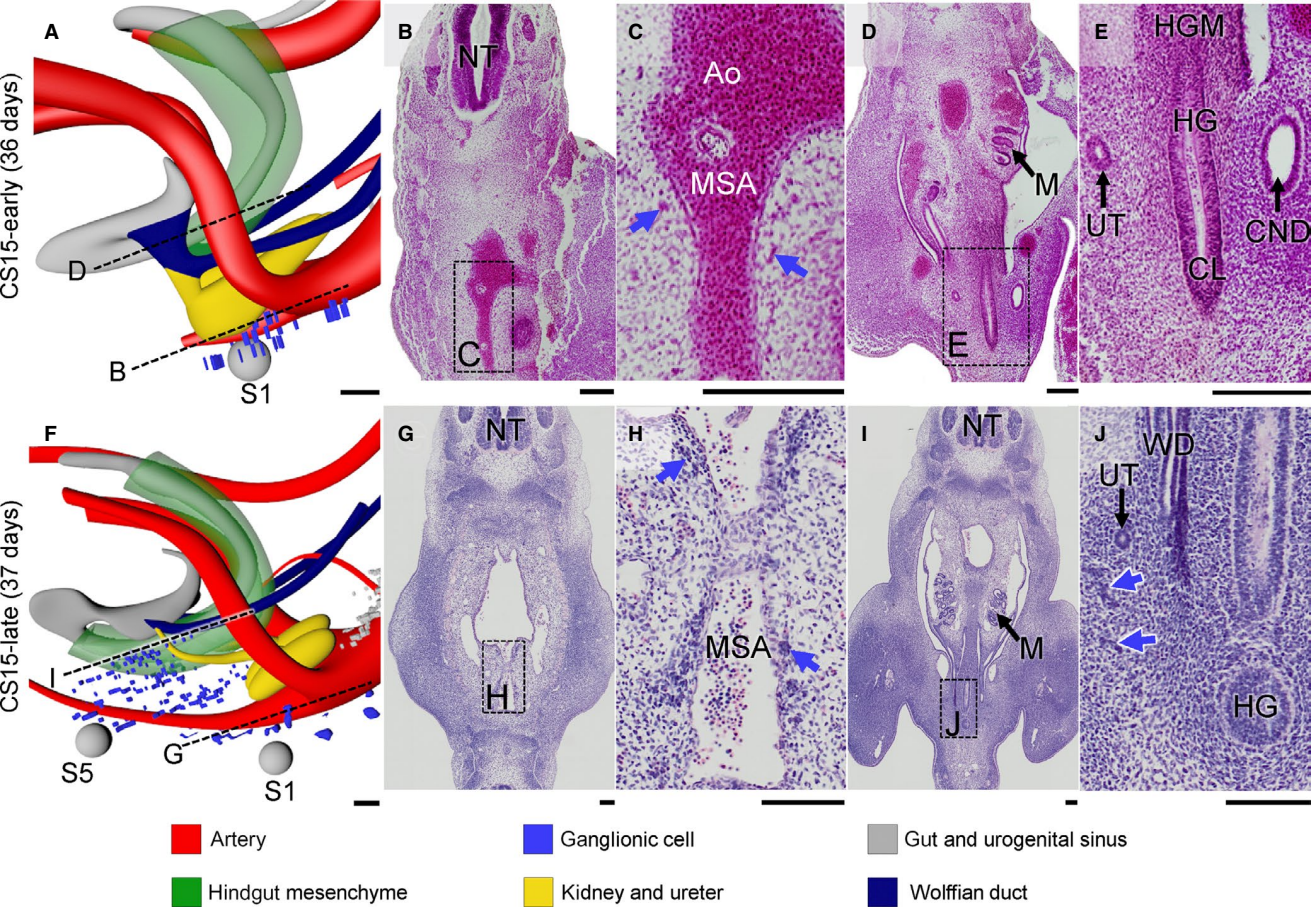
Migration of neural crest‐derived ganglionic cells at Carnegie stage (CS)15. Panels a and f show side views of the lesser pelvis of CS15‐early and CS15‐late embryos, respectively. Panels b, d, g and i show histological sections and panels c, e, h and j magnifications of the respective boxes. At CS15‐early (panel a) ganglionic cells (blue dots in panel a, blue arrows in panel c) migrate ventrally to the para‐arterial region at level S1 (grey sphere). One day later (CS15‐late) such ganglionic cells have migrated further ventrally towards the hindgut (blue arrows in panel j) and further caudally to level S5. The median sacral artery is considered as the caudal extension of the dorsal aorta. Bars = 100 µm. Ao, aorta; CL, cloaca; CND, common nephric duct; HG, hindgut; HGM, hindgut mesenchyme; M, mesonephros; MSA, median sacral artery; NT, neural tube; UT, ureter; WD, wolffian duct. [Colour figure can be viewed at wileyonlinelibrary.com]

**FIGURE 3:**
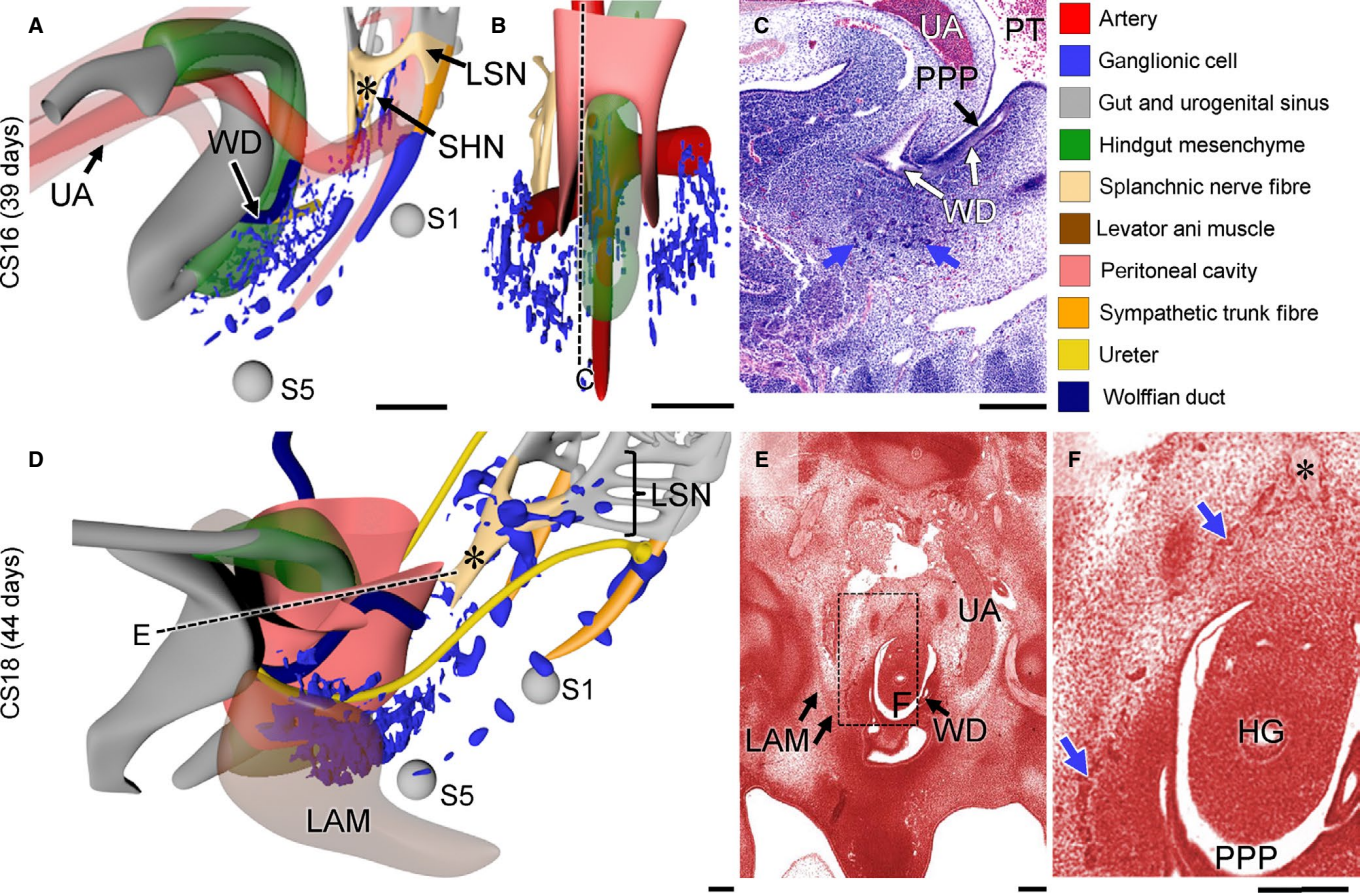
Appearance of the inferior hypogastric ganglionic cell cluster (IHGC). Panels a and d show side views of the lesser pelvis of Carnegie stage (CS)16 and CS18 embryos, respectively. Panel b shows a frontal view of the CS16 reconstruction to show that both IHGCs contact each other dorsal to the hindgut. Panels C and E show histological sections and panel F a magnified view of the box in e. At CS16 the bulk of ganglionic cells is present as bilateral clusters (blue dots in panels a, b and blue arrows in panel c) that have migrated ventrally as far as the entrance of the Wolffian ducts. The levator ani muscle formed at CS18 as a mesenchymal condensation lateral to IHGCs. LAM, levator ani muscle; LSN, lumbar splanchnic nerve; PPP, pelvic peritoneal pouch; PT, peritoneal cavity; SHN, superior hypogastric nerve; UA, umbilical artery; WD, wolffian duct. Bars = 200 µm. [Colour figure can be viewed at wileyonlinelibrary.com]

**FIGURE 4:**
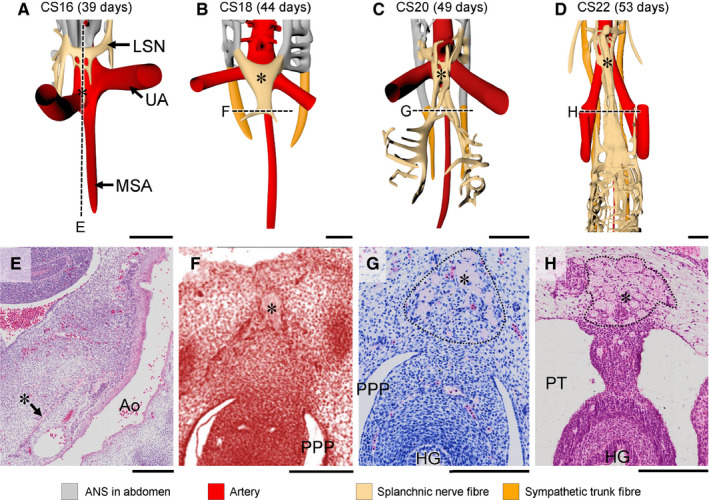
The superior hypogastric nerve is a caudal extension of the lumbar splanchnic nerves. Panels a–d show frontal views of nerve fibres and arteries in Carnegie stage (CS)16 ‐CS22 embryos, while panels e–h show histological sections along the black dotted lines in panels a–d. At CS16 nerve fibres of the lumbar splanchnic nerves (LSN) extended caudally across the bifurcation of the umbilical arteries as the superior hypogastric nerve (SHN; asterisks in panels a, e). The SHN extended caudally as a single trunk that bifurcated caudally (asterisks in panels b, f) at CS18. From CS20 onwards the single SHN trunk split into multiple nerve fibres (asterisks in panels c, d, g, h, with the contour of the original nerve outlined as dots), that are known as the superior hypogastric plexus. Ao, aorta; MSA, median sacral artery; PPP, pelvic peritoneal pouch; PT, peritoneal cavity; UA, umbilical artery. a–d, bars = 500 µm; e–h, bars = 200 µm. [Colour figure can be viewed at wileyonlinelibrary.com]

In the pelvic area, NCCs were first found dorsolaterally to the median sacral artery near level S1 in CS15‐early embryos (~36 days of development; blue dots in Figure 2A and blue arrows in Figure 2C). Topographically, these ganglionic cells were a caudal continuation of similarly located ganglionic cells in the lumbar region and, accordingly, passed the bifurcating umbilical arteries dorsally (Figure S2A, CS14‐early). At CS15‐late (~37 days), the dorsolateral NCCs had extended their presence to level S5 (blue dots in Figure 2F and blue arrows in Figure 2H,J). The most cranial portion of these cells began to consolidate as ganglia of the sympathetic trunk along the median sacral artery (blue arrows in Figure 2H). Furthermore, many scattered cells had now accumulated ventrally to the median sacral artery and laterally to the hindgut, where they occupied on both sides a triangular area with its base dorsally between S1 and S5, and its apex ventrally near the entrance of the common nephric portion of the Wolffian ducts into the urogenital sinus. These ganglionic cells were found caudal to the umbilical arteries and therefore did not extend into the abdominal cavity (Figure 2F). Their ventral extension was situated laterally to the pelvic pouch of the coelomic cavity. This pelvic pouch located in the urorectal septum and surrounded the hindgut ventrally and laterally down to the cloaca (Kruepunga *et al*. 2018; Figure S2B), so that the ventrolateral ganglionic cells were not in direct contact with the wall of the hindgut. At CS16 (~39 days of development; Figure 3A–C), ganglionic cells had advanced further ventrally along the lateral side of the pelvic pouch to form the left‐ and right‐sided, sagittally oriented inferior hypogastric ganglionic cell clusters (IHGCs; Figure 3B). The presence of the pelvic pouch explains why both IHGCs persisted as separate entities throughout subsequent development. More cranially, in a plane perpendicular to the notochord at level S1 and through the junction of mid‐ and hindgut, cells of the IHGCs that remained located mediodorsally to the hindgut connected to the well‐developed superior hypogastric nerve (SHN in Figure 3A; asterisk in Figure 4A), which is the common caudal continuation of the left‐ and right‐sided lumbar splanchnic nerves along the superior rectal branch of the IMA. Strikingly, no caudal extensions of ganglionic cells toward the cloaca were seen. Ganglionic cells dorsolaterally to the median sacral artery had now aggregated to such an extent that they could be identified as the sacral sympathetic trunk (Figure 3A).

**FIGURE 5:**
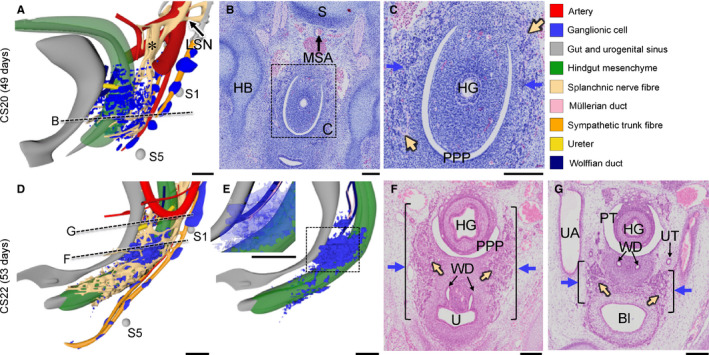
Extension and incorporation of nerve fibres mark the formation of the inferior hypogastric plexus. Panels a, d, e show oblique views of the lesser pelvis at Carnegie stage (CS)20 (a) and CS22 (d, e). Panels B and F show histological sections and their magnified views (c, g) indicated by rectangles. At CS20, the lumbar splanchnic nerve (asterisk in panel a) continued caudally as the superior hypogastric plexus that extended into the IHGCs (beige and blue arrows in panel c, respectively). At CS22 the inferior hypogastric ganglionic cell clusters (blue dots in panel e and blue arrows in panels f) were largest at the level of the entrance of the Wolffian duct into the urogenital sinus. The inferior hypogastric plexus formed cranial extensions along bladder neck (brackets in panels f, g) and caudal extensions into the perirectal mesenchyme (Figure 5d, e). Note that the mesenchyme nor the nerves reach the dorsal cloaca (grey tip extending beyond hindgut mesenchyme). Bl, bladder; HB, hip bone; HG, hindgut; LSN, lumbar splanchnic nerve; MSA, median sacral artery; PPP, pelvic peritoneal pouch; PT, peritoneal cavity; S, sacrum; U, urogenital sinus; UA: umbilical artery; UT, ureter; WD, wolffian duct. a, d, e bars = 500 µm; b, c, f, g, bars = 200 µm. [Colour figure can be viewed at wileyonlinelibrary.com]

At CS18 (~44 days of development), the cell density in the IHGCs had further increased (Figure 3D) and came to resemble the preaortic ganglia seen more cranially (Kruepunga *et al*., in press). Cranially, the continuity between the left and right ganglionic‐cell clusters and the large medial superior hypogastric nerve was now firmly established (Figure 3D–F). While the dorsal boundary of the IHGCs retained its position dorsolateral to the hindgut, its ventral boundary advanced into the mesenchymal niche between the pelvic coelomic pouch and urogenital sinus (Figure 3D–F). Laterally, these ganglionic cells were flanked by the levator muscle, which had become identifiable as a well‐defined condensation of mesenchyme flanking the gut between S2 and S5 (LAM; transparent brown in Figure 3D). At CS20 (~49 days of development), the developmental events described for CS18 had continued to advance, with a major feature being a quantitative increase in the density of ganglionic cells in both IHGCs (Figure 5A–C). In CS22 embryos (~53 days of development; Figure 5D–G), the left and right IHGCs were still separate, but each cluster had fragmented (blue dots in panels 5D and E). Its middle portion, which occupied the niche between the urogenital sinus ventrally and pelvic coelomic pouch at the level of the entrance of the Wolffian and Müllerian ducts into the urogenital sinus dorsally, had evolved as the biggest and densest (blue dots in panel 5E, blue arrows in panels 5F and G). Because each IHGC now also incorporated nerve fibres, it could be labelled the inferior hypogastric or pelvic plexus (blue dots and beige network in Figure 5D). The more cranial and caudal portions of the IHGCs formed small clusters near the ureteric entrance into the bladder and around the lower part of the rectum, respectively (Figure 5D). Interestingly, the caudal extension of the IHGC reached the rectal wall caudal to the pelvic coelomic cavity, which at this stage had started to regress in a cranial direction (Hikspoors *et al*. 2019).

### 3.3 Formation of the nerve fibre network of the inferior hypogastric plexus

Nerve fibres were first observed in the lesser pelvis at CS16, when the superior hypogastric nerve formed as a median continuation of both lumbar splanchnic nerves along the superior rectal artery. This nerve formed cranial to the bifurcation of the umbilical arteries, but began to extend caudally across the bifurcation towards the cranial part of both IHGCs described in the previous section (asterisks in Figure 4A,E). In CS18 embryos, the superior hypogastric nerve had increased in diameter and length, so that it now passed the aortic bifurcation. This single nerve trunk (asterisks in Figure 4B,F) bifurcated just caudal to the umbilical arteries to form both hypogastric nerves that each connected to the corresponding IHGC. Nerve fibres were still absent from the IHGCs at this stage (Figure 3D–F). Subsequently (CS20), the superior hypogastric nerve at the intestinal base of the dorsal mesentery split into a bundle of smaller nerves that began to resemble the superior hypogastric plexus (asterisks in Figure 4C,G). Fragmentation of the nerve into a plexus continued through CS22 (asterisks in Figure 4D,H).

**FIGURE 6:**
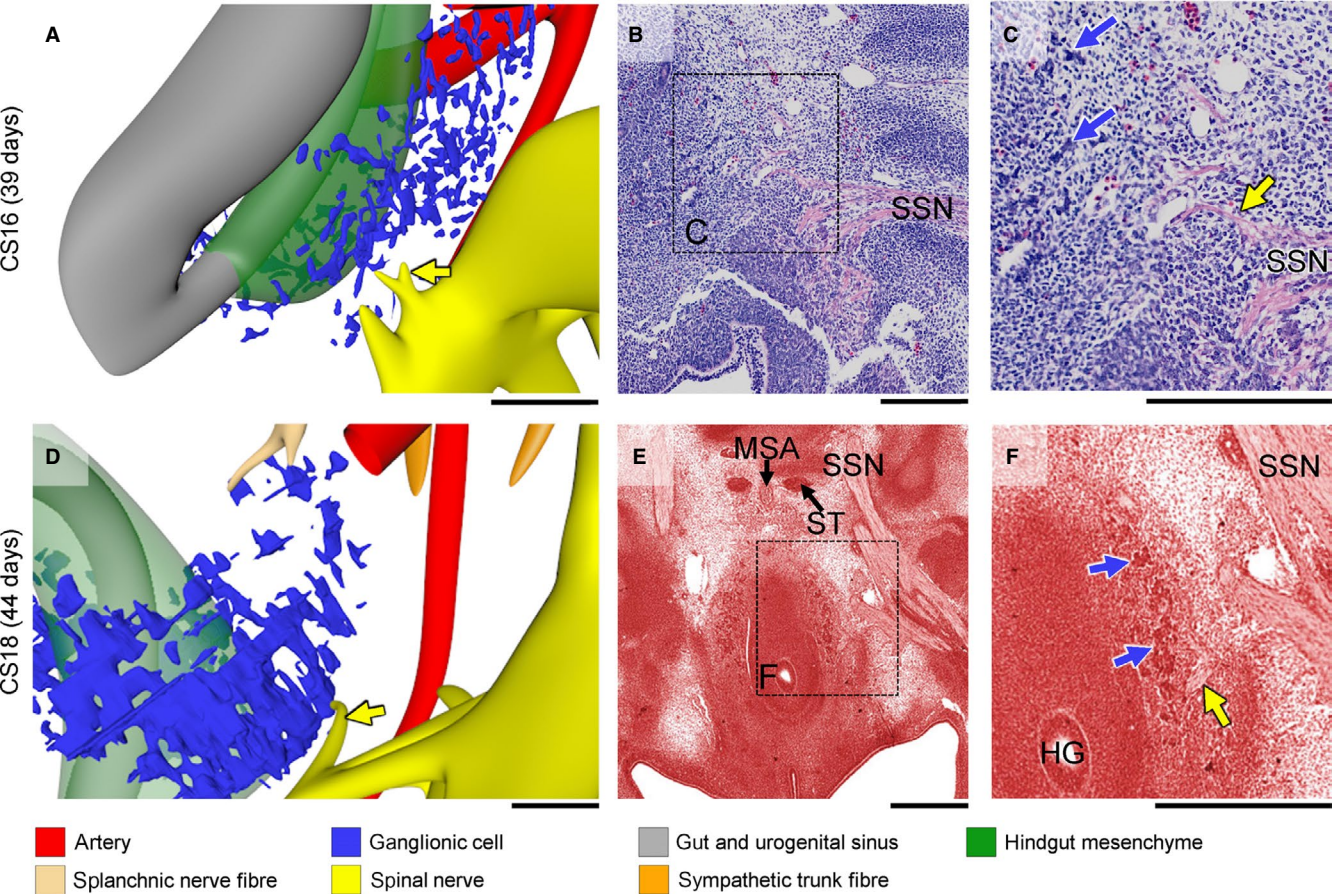
Appearance of the pelvic splanchnic nerves. Panels a, d show the pelvic splanchnic nerves as small medial branches (yellow arrows) of the sacral spinal nerves, while panels b, c, e, f show histological sections and magnified views indicated by rectangles. At Carnegie stage (CS)16 the pelvic splanchnic nerves had not yet reached the inferior hypogastric ganglionic cell clusters (blue dots in panel a and blue arrows in panel c), whereas at CS18 they had (yellow arrows in panels d and f). MSA, median sacral artery; SSN, sacral spinal nerve; ST, sympathetic trunk. a–d bars = 200 µm; e, f bars = 500 µm. [Colour figure can be viewed at wileyonlinelibrary.com]

**FIGURE 7:**
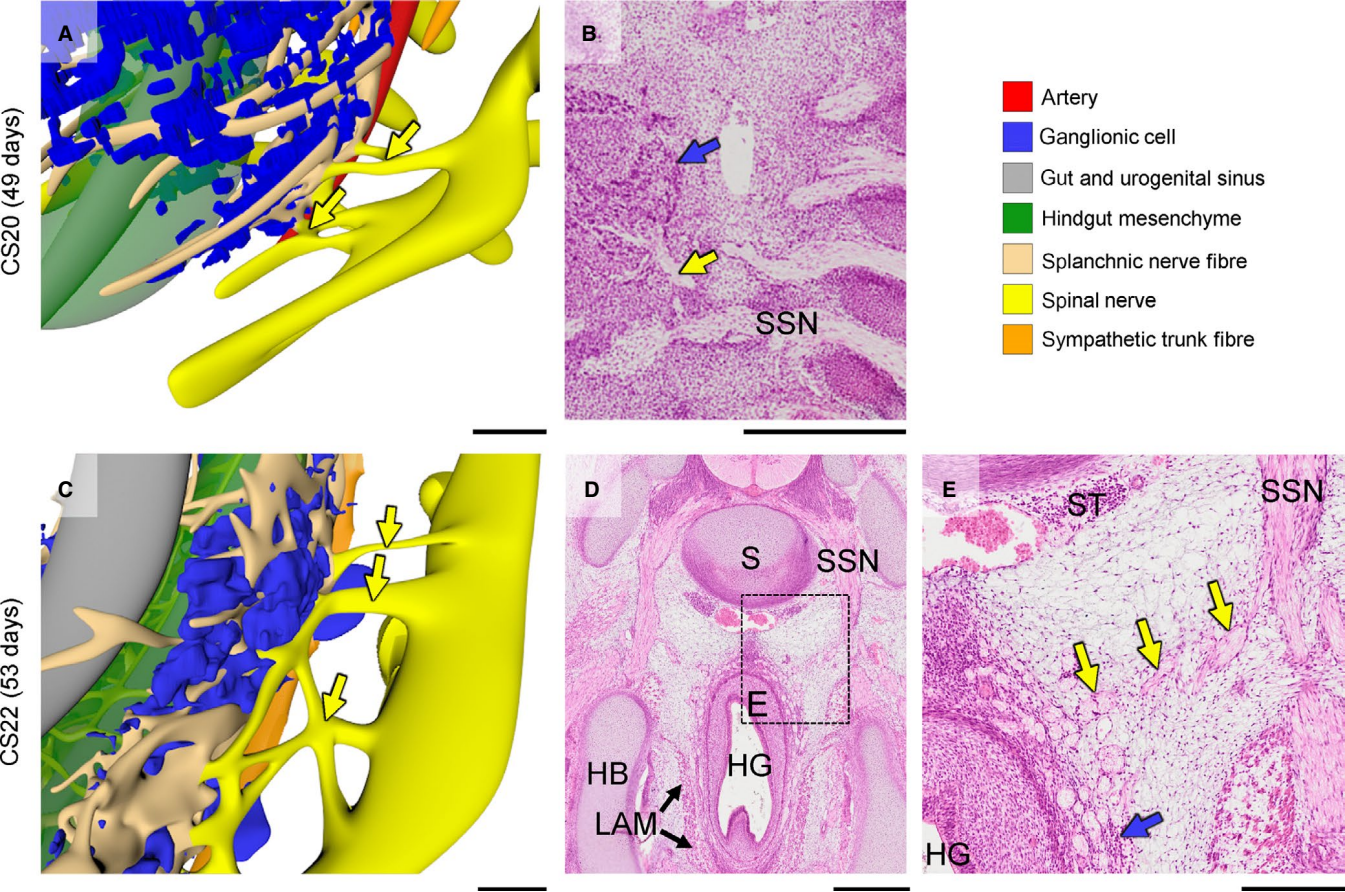
Extension of the pelvic splanchnic nerves. Panels A, C show pelvic splanchnic nerves, while panels b, d, e show histological sections and a magnified view (rectangle). At Carnegie stage (CS)20 and CS22 pelvic splanchnic nerves (yellow arrows in panels b and e) had penetrated into the inferior hypogastric ganglionic cell clusters (IHGCs; blue dots in panels a, c and blue arrows in panels b and e). HB, hip bone; HG, hindgut; LAM, levator ani muscle; S, sacrum; SSN, sacral spinal nerve; ST, sympathetic trunk. a, c, e, bars = 200 µm; b, d, bars = 500 µm. [Colour figure can be viewed at wileyonlinelibrary.com]

**FIGURE 8:**
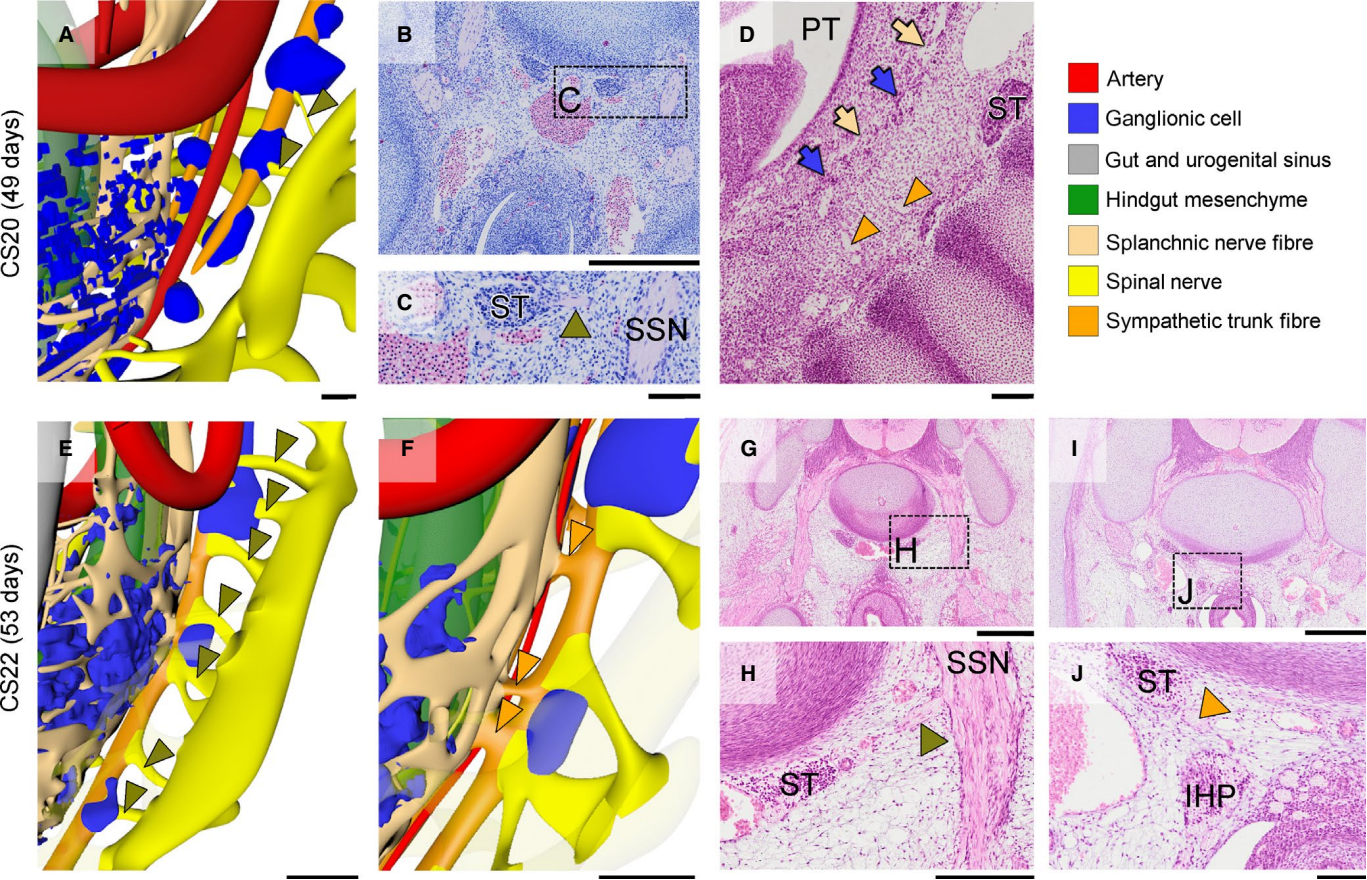
Formation of sacral communicating branches and splanchnic nerves. Panels a, e, f show connections between the sympathetic trunk ganglia and the sacral spinal nerves (communicating branches; olive green arrowheads in a, e) and with the inferior hypogastric plexus (sacral splanchnic nerves; orange arrowheads in f). Panels b–d and g–j show histological sections and their magnified views (rectangles). The communicating branches in panels c and h are indicated with olive‐green arrowheads, while the sacral splanchnic nerves are indicated with an orange arrowhead (panel j). IHP, inferior hypogastric plexus; PT, peritoneal cavity; SSN, sacral spinal nerve; ST, sympathetic trunk. Bars a, e, f, h = 200 µm, b, g, i = 500 µm c, d, j, k = 100 µm. [Colour figure can be viewed at wileyonlinelibrary.com]

Concomitantly, medial branches of sacral spinal nerves S2–4, known as the pelvic splanchnic nerves, extended medially towards the IHGCs at CS16 (yellow arrows in Figure 6A,C). The pelvic splanchnic nerves reached the IHGCs at CS18 (yellow arrows in Figure 6D,F) and lined up with nerves in the IHGCs in CS20 embryos (Figure 7A). The resulting network of fibres inside the left and right IHGCs had, therefore, transformed these clusters into the left and right inferior hypogastric plexuses (Figure 5A‐C). Furthermore, nerve fibres from the sympathetic trunk had started to extend ventrally as the sacral splanchnic nerves and connected to the inferior hypogastric plexus at CS22 (orange arrowheads in Figure 8D,F,J).

**FIGURE 9:**
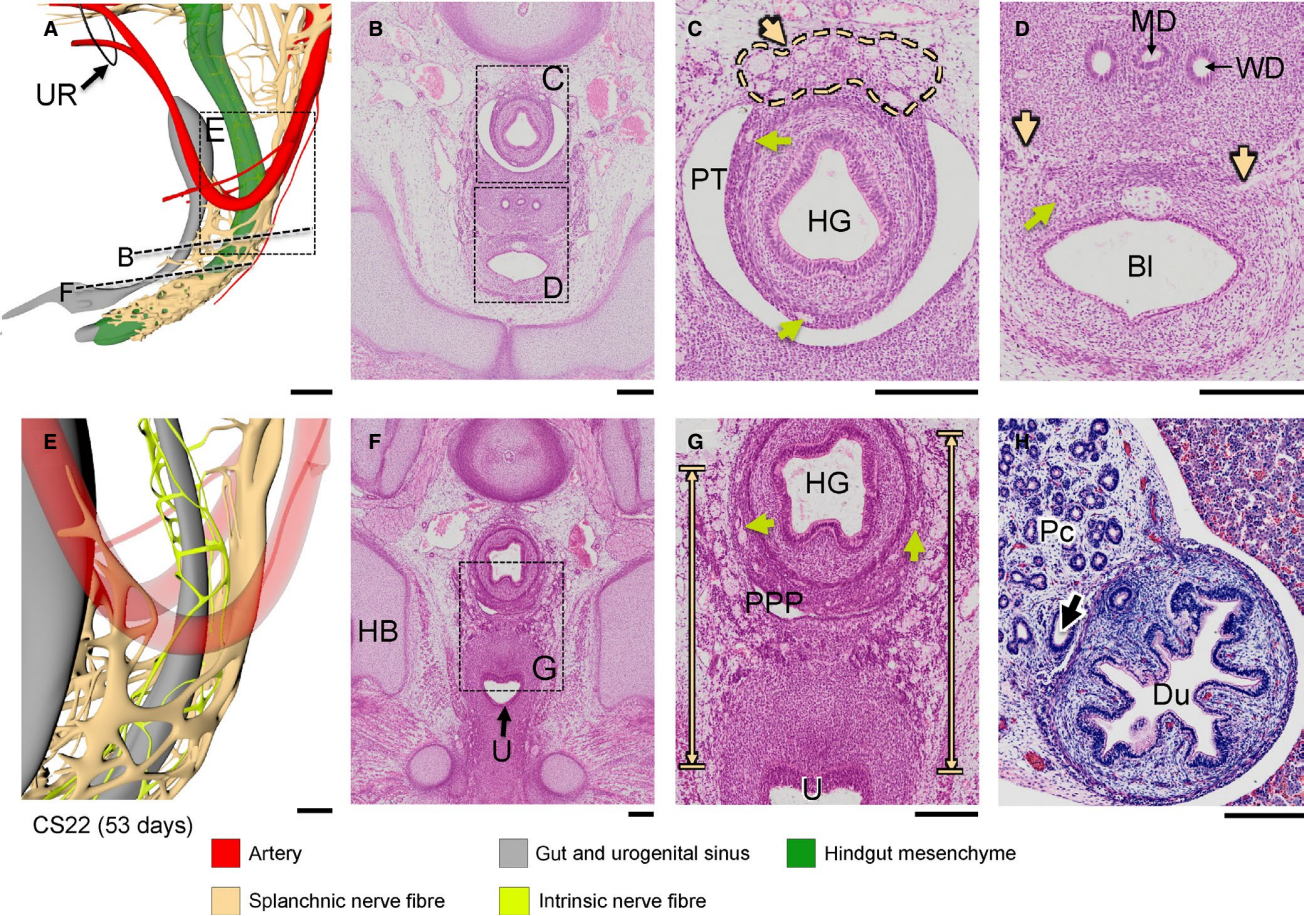
Connection of extrinsic and intrinsic enteric nerve fibres in the lesser pelvis. Panels a, e show nerve fibres in the lesser pelvis and a magnified view (rectangle). Panels b–d and f–g show histological sections from the levels indicated by dotted lines in panel a and magnified views (rectangles). Extrinsic enteric nerve fibres (beige arrows) extend into the mesenchymal cuffs of the hindgut, where intrinsic nerve fibres (light green arrows) form an intrinsic nervous network (panels c, g). In contrast, such well‐developed nerves are near absent in the duodenum (panel h; arrow indicates common bile duct). Bl, bladder; Du, duodenum; HB, hip bone; HG, hindgut; IHP, inferior hypogastric plexus; MD, Müllerian duct¶; Pc, pancreas; PPP, pelvic peritoneal pouch; PT, peritoneal cavity; SSN, sacral spinal nerve; ST, sympathetic trunk; U, urogenital sinus; UR, umbilical ring; WD, Wolffian duct. Bars a = 500 µm, b–g = 200 µm. [Colour figure can be viewed at wileyonlinelibrary.com]

The 8th embryonic week (CS20–CS23) was characterized by a pronounced increase in size and density of the network of nerve fibres in the inferior hypogastric plexuses. At the end of the 8th week, there were three groups of nerve fibres associated with the inferior hypogastric plexus: hypogastric nerves originating in the lumbar splanchnic nerves (segments L1, 2) and accompanying the superior rectal artery (Figure 4), pelvic splanchnic nerves originating in the sacral spinal nerves (segments S2–4; Figures 6 and 7), and sacral splanchnic nerves originating in the sympathetic trunks (originating from segments L1, 2, but passing through the sacral sympathetic trunk; Figure 8 and Figure S3C). The nerve fibres of the inferior hypogastric plexus extended cranially to the developing muscular layer of the bladder fundus as vesical plexus and caudally to the distal hindgut (but not dorsal cloaca) as rectal plexus, while tiny fibres near the entrance of the Wolffian and Müllerian ducts extended into the wall of the urogenital sinus as uterovaginal or prostatic plexus (Figure S3C). The bifurcation of the superior hypogastric plexus into left‐ and right‐sided hypogastric nerves was located at the level of S1. In addition, many left–right connections between both sides of the inferior hypogastric plexus had formed on the dorsal side of the distal hindgut. The sacral spinal nerves clearly had medial extensions (pelvic splanchnic nerves) to the inferior hypogastric plexus (yellow arrows in Figure 7C,E) and ventral extensions that formed the pudendal nerve (Figure S3C). In addition, extrinsic enteric nerve fibres (beige arrows) within the inferior hypogastric plexus had connected intrinsic enteric nerve fibres (light green arrows) in the urogenital and hindgut mesenchyme (Figure 9). While the network of nerve fibres in the wall of the hindgut was well defined (Figure 9C), such a network of nerve fibres was not present in the wall of, for example, the duodenum (Figure 9H). At CS22 and CS23, no differences in pelvic innervation between male and female embryos were apparent.

## 4 DISCUSSION

**FIGURE 10:**
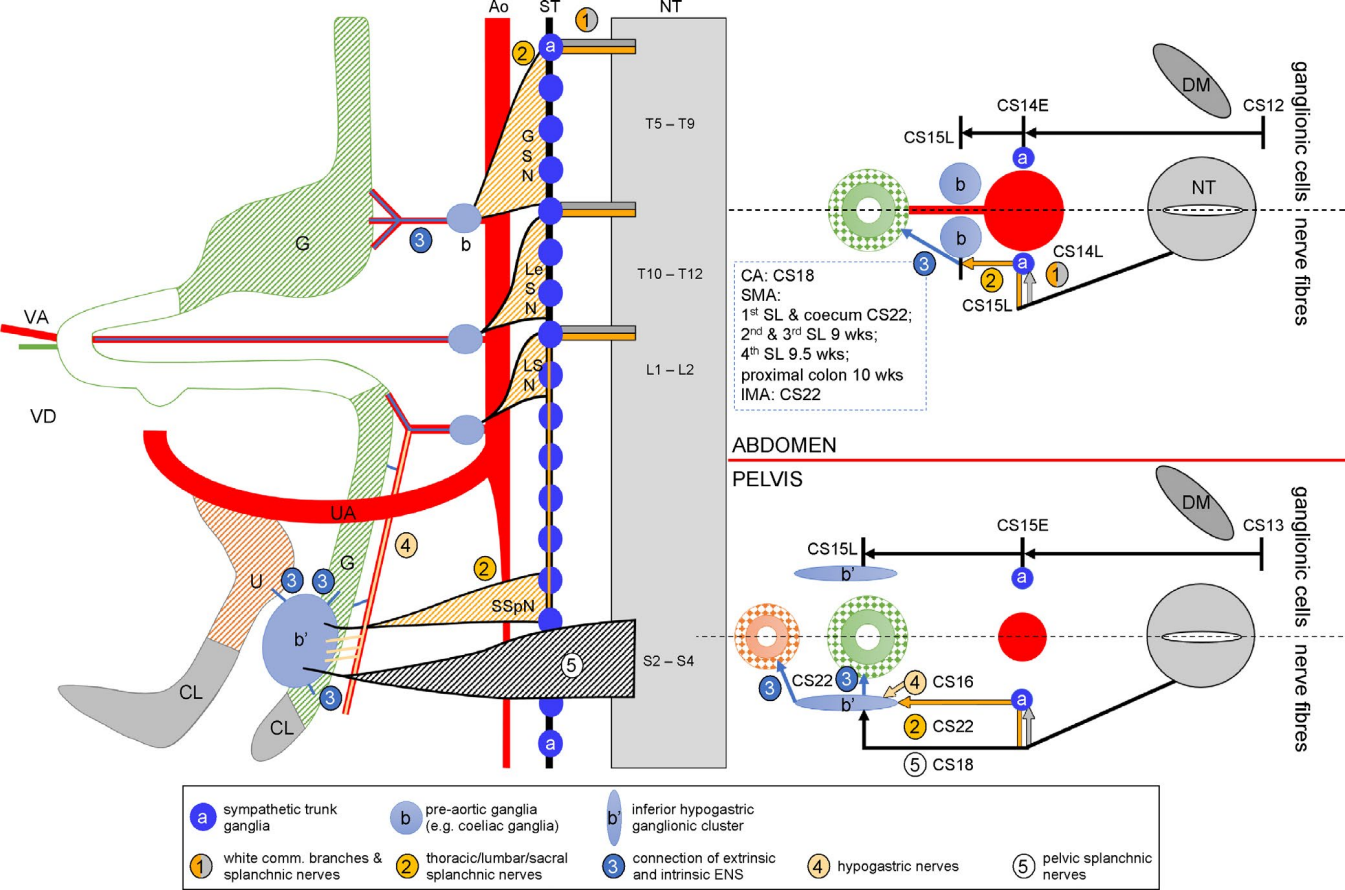
Preganglionic innervation of pre‐aortic plexuses; comparison of the thoracolumbar and pelvic areas. The left panel shows a scheme of the ventral branches of the aorta and median sacral artery (red), the gastrointestinal tract (green) and cloaca (grey). The scheme shows the preganglionic autonomic connections (coded in hatched orange: greater splanchnic nerves (GSN; T5‐T9); lesser and least splanchnic nerves (LeSN; T10‐T12); and lumbar splanchnic nerves (LSN; L1‐L2) to the pre‐aortic ganglia (grey‐blue ovals 'b'). Preganglionic nerves pass the sympathetic trunk (blue) without synapsing. The inferior hypogastric plexus ('IHP' in large grey‐blue oval) is innervated by the superior hypogastric nerve ('4'); the sacral splanchnic nerves (SSpN; also coded in hatched orange); and the pelvic splanchnic nerves ('5'; S2‐4; coded in hatched black). The right panel compares the timeline of the migration of the ganglionic cells (upper half of schemes) and that of the nerve fibres (lower half of schemes) in the abdomen (upper scheme) and pelvis (lower scheme). Both schemes show that the ganglionic cells migrate equally fast to the pre‐arterial locations in the abdomen and pelvis, but that it takes the nerve fibres in the pelvic area longer to reach their targets. Ao, aorta; CL, cloaca; DM, dermomyotome; G, gut; NT, neural tube; ST, sympathetic trunk; U, urogenital sinus; UA, umbilical artery. [Colour figure can be viewed at wileyonlinelibrary.com]

**FIGURE 11:**
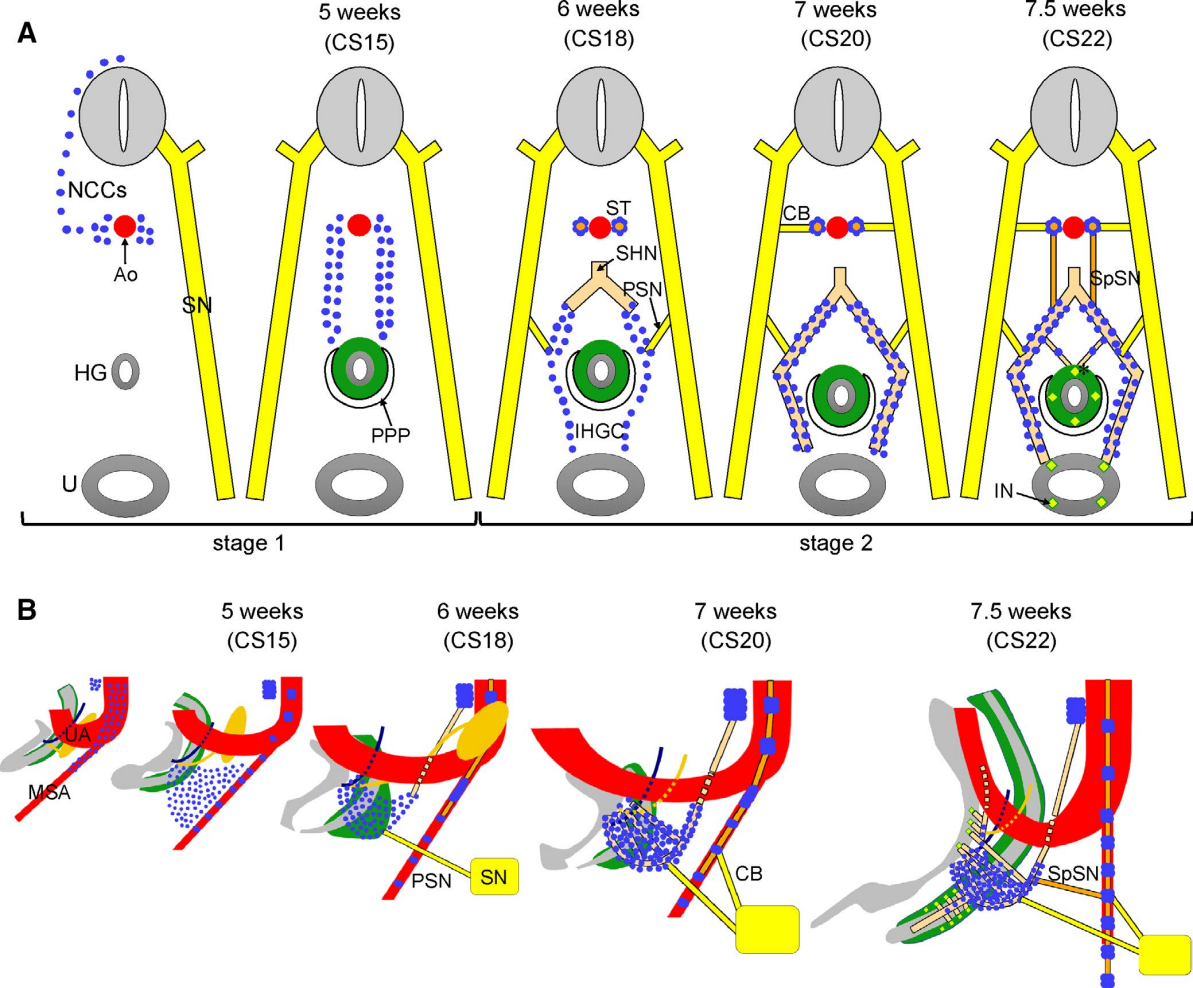
Timeline of neural crest‐cell migration to ganglionic cell clusters in, and preganglionic innervation of the inferior hypogastric plexus. Panel A shows schematic transverse sections at different developmental timepoints. The development of the inferior hypogastric plexus can be divided into (1) migration of neural crest cells (blue dots) towards their para‐arterial [Carnegie stage (CS)14 and CS15] and pre‐arterial positions (CS15 and CS16); and (2) association of nerve fibres with the inferior hypogastric ganglionic cell cluster. The first nerve to arrive is the superior hypogastric nerve at CS16, followed by the pelvic splanchnic nerves at CS18 and the sacral splanchnic nerves at CS22. By that time small nerve fibres had entered the gut wall via the dorsal mesentery of the hindgut and contacted intrinsic enteric nerves (asterisk in far right subpanel). Ao, aorta; CB, communicating branch; HG, hindgut; IHGC, inferior hypogastric ganglionic cluster; IN, intrinsic nerve fibre; MSA, median sacral artery; NCC, neural crest cell; PPP, pelvic peritoneal pouch; PSN, pelvic splanchnic nerve; SHN, superior hypogastric nerve; SN, sacral nerve; SSpN, sacral splanchnic nerve; ST, sympathetic trunk; U, urogenital sinus; ST, sympathetic trunk; UA, umbilical artery. [Colour figure can be viewed at wileyonlinelibrary.com]

We studied the development of the extrinsic innervation in the lesser pelvis to illustrate and explain its spatiotemporal population with autonomic ganglionic cells and nerve fibres (for a pictorial summary, see Figures 10 and 11).

### 4.1 Boundaries of the lesser pelvis

The hip bone and sacrum are well established lateral boundaries of the lesser pelvis and can also be used as such in embryos, but landmarks for the cranial and caudal boundaries of the lesser pelvis are less evident. In the present study, we have identified the umbilical arteries as landmarks for its cranial boundary. Their position corresponds with the cranioventral brim of the hip bones. Two landmarks reveal the developmental changes in the hip and the lesser pelvis: the apparent rotation of the so‐called 'pubococcygeal line' between the inferior rim of the pubic symphysis and the caudal end of the sacrum (Fielding, 2002), and the changing proximal course of the umbilical arteries both show that the caudal spine and its surroundings straighten by the unfolding of their previously pronounced kyphosis (Figure 1; Kruepunga *et al*. 2018). Based on the pronounced increase in width of the dorsal mesentery, the transition between mid‐ and hindgut co‐localizes with the position of the umbilical arteries. This finding indicates that the transition between the herniating and non‐herniating parts of the colon has to be located at the transition of sigmoid colon into the rectum.

The caudal boundary of the lesser pelvis is formed by the muscles of the pelvic floor (external urethral sphincter, levator ani and coccygeus), of which the sphincter and coccygeus muscles are remarkably well developed in 8‐week embryos (see also Tichý, 1989; Koch and Marani, 2007). The boundary function of the LAM is also clear from the course of the nerves surrounding it: nerve fibres medial to the LAMs extend only inside the lesser pelvis, whereas nerve fibres on its lateral side (the pudendal nerve) extend to the mesenchymal structures outside the lesser pelvis (Figure S3C).

### 4.2 Similarities and differences in the developing extrinsic innervation of the gut in the abdomen and lesser pelvis

A number of instructive schematic accounts of the development of the autonomic enteric innervation of the thorax and abdomen are available, in particular for its intrinsic component (e.g. Lake and Heuckeroth, 2013; Saito and Takahashi, 2015; Espinosa‐Medina *et al*. 2017; Dyson *et al*. 2018; Simkin *et al*. 2019) and, albeit to a lesser extent, also for its extrinsic component (Hatch and Mukouyama, 2015; Uesaka *et al*. 2016; Kruepunga et al., in press). However, graphic accounts of the ENS in the lesser pelvis (e.g. Wang *et al*. 2011) are rare. Although the contribution of Schwann cell precursors (SCPs) to the chromaffin cells of the autonomic nervous system in the abdomen was recently estimated to be ~80% (Furlan *et al*. 2017; Kastriti *et al*. 2019) and that to the intrinsic neurons of the colon ~20% (Uesaka *et al*. 2015), we do not know at present if, and if so to what extent, SCPs contribute to the IHGCs. In addition, the identity of the sacral outflow was recently questioned and reclassified as sympathetic, that is, similar in structure and developmental origin to the thoracic and lumbar autonomic nervous system (Espinosa‐Medina *et al*. 2016). We, therefore, compared the timing and sequence of appearance and differentiation of the building blocks (ganglionic cells and nerve fibres) that formed the abdominal and pelvic pre‐aortic ganglia in human embryos.

#### 4.2.1 Migratory timeline of neural crest‐derived ganglionic cells

Cell tracing studies showed that the ganglionic cells in the lesser pelvis arise from sacral NCCs in mammalian embryos (Serbedzija *et al*. 1991; Kapur, 2000; Anderson *et al*. 2006; Wang *et al*. 2011; Wiese *et al*. 2017). In human embryos, the thoracolumbar neural crest emerges during CS12 and the sacral neural crest during CS13 (Figure 10), that is, with a ~2‐day delay (O’Rahilly and Müller, 2007). If the dorsal aorta and median sacral artery can serve as homologous landmarks, NCCs at both locations first aggregate laterally to the dorsal aorta (para‐aortic cluster) and then ventrally to the aorta (pre‐aortic cluster) (Figures 10 and 11). Thoracolumbar NCCs begin to migrate to the para‐aortic region during CS13 (~31 days) and arrive at CS14‐early (~33 days). These NCCs either settle there to form sympathetic ganglia 3 days later [CS15‐early (~36 days)] or continue migration to arrive at the pre‐aortic region 4 days later [CS15‐late (~37 days)] and form the pre‐aortic plexuses. NCCs in the lesser pelvis arrive in the para‐arterial region only at CS15‐early (~36 days) to form sympathetic ganglia. However, the NCCs that continue to migrate ventrally need just ~1 day to reach the pre‐arterial region at CS15‐late (~37 days) and form the IHGC (equivalent to pre‐aortic ganglia) (Figure 11). This comparison shows that the timeline of the migration of ganglionic cells in the thoracolumbar and sacral region differs, but that the migratory pathway and time of arrival at their destination are very similar.

#### 4.2.2 Differences in innervation of the neural crest‐derived ganglionic‐cell aggregates

The pre‐aortic plexuses in the thoracolumbar region are irregularly shaped median masses of neural crest‐derived ganglionic cells that surround the roots of the ventral branches of the dorsal aorta. In addition, agglomerates of the SCP subgroup of NCCs are found in association with the coeliac and inferior mesenteric plexuses as the (developing) chromaffin cells of the adrenal medulla and para‐aortic bodies, respectively (Furlan *et al*. 2017; Kastriti *et al*. 2019). These two agglomerates differ in that the chromaffin cells of the adrenal medulla are positioned dorsolaterally to the coeliac plexus, whereas those of the para‐aortic bodies touch or merge in the midline (Figure S5A,B), with very few para‐aortic bodies found distal to the umbilical bifurcation (Coupland, 1952, Kruepunga *et al*., in press). The preganglionic nerve fibres that innervate the pre‐aortic ganglionic‐cell agglomerates arrive at CS15 late (~37 days of development; Figure 10). The IHGCs form at the same developmental stage as, and are contacted only slightly later (CS16; ~39 days) by the superior hypogastric nerve than the inferior mesenteric cluster by the lumbar splanchnic nerves (Kimmel and McCrea, 1958; Arango‐Toro and Domenech‐Mateu, 1993). In fact, our reconstructions show that the superior hypogastric nerve develops as an unpaired mediocaudal extension of the lumbar splanchnic nerves along the superior rectal artery (CS16–20), which explains the common origin of the lumbar splanchnic and superior hypogastric nerves in segments L1 and L2. At CS20, the superior hypogastric nerve has split up into several and at CS22 into many separate nerve fibres. This remarkable splitting of a compact nerve into a distributed plexus may well explain its morphological variability and many alternate names in the adult (Davis, 1934).

Apart from the hypogastric nerves, the IHGC has two additional sources of preganglionic innervation, of which the pelvic splanchnic nerves arrive at CS18 (~44 days) and the sacral splanchnic nerves only at CS22 (~53 days). Phenotypically and developmentally, the prenatal pelvic splanchnic nerves were recently characterized as sympathetic rather than parasympathetic (Espinosa‐Medina *et al*. 2016), so of comparable phenotype to the hypogastric and sacral splanchnic nerves. Our findings refine earlier timelines in human embryos (Kuntz, 1952; Kimmel and McCrea, 1958; Arango‐Toro and Domenech‐Mateu, 1993) and better allow comparison with data obtained in experimental animal models, such as mice. However, cause(s) and consequence(s) of this phased innervation by phenotypically similar preganglionic nerves remain to be clarified.

#### 4.2.3 Differences in topography of the abdominal and pelvic plexuses

Compared to the midline pre‐aortic abdominal ganglia, the bilateral presence of the inferior hypogastric plexuses is striking. This pronounced topographic difference can be ascribed to the pelvic pouch, a caudal extension of the peritoneal cavity between the hindgut and the urogenital sinus that extends down to the muscular pelvic floor. The temporary presence of this narrow pouch in human embryos was first described by Cunéo and Veau, 1899 and confirmed by Tobin and Benjamin, 1945, and Uhlenhuth *et al*. 1948. It surrounds the ventral and lateral sides of the hindgut, leaving a wide dorsal mesentery. The pelvic pouch, like the nearby vaginal process in the groin, obliterates exclusively in hominids and not in quadrupeds. This process proceeds from caudal to cranial and begins in the 8th week (Kruepunga *et al*. 2018; Hikspoors *et al*. 2019) to reach its definitive position at ~11 weeks development (Tobin and Benjamin, 1945; Uhlenhuth *et al*. 1948; Fritsch, 1988). Obliteration of the pouch may protect bipedal hominids from rectal prolapse, which is quite common in quadrupeds (Pettan‐Brewer and Treuting, 2011). Accordingly, a straight course of the sigmoid colon with a correspondingly short mesentery (both embryonic features) often co‐occur with a persisting pelvic coelomic pouch and enterocele (Baessler and Schuessler, 2006). The presence of a dorsal mesentery allows extrinsic nerve fibres, which guide ganglionic cells towards and into the wall of the hindgut, to enter the wall of the hindgut dorsally (Erickson *et al*. 2012), but the ganglionic cells that eventually form the inferior hypogastric cluster, have to migrate laterally to the pelvic pouch, ureters and Wolffian ducts towards their future location (Figures 2, 3, 4). Even though present only temporally, the pelvic pouch, together with the dense mesenchyme surrounding the urogenital sinus, prevent the left‐ and right‐sided ganglionic cells from forming a single midline structure. Denonvilliers’ fascia will form at the site of adhesion and fusion of the mesothelial layers of the pelvic pouch. This configuration also explains why, if possible, dissection anterior (ventral) to Denonvilliers’ fascia is to be preferred over posterior dissection during a total mesorectal excision (Fang *et al*. 2019).

### 4.3 Limitations of the study

The present study provides detailed reconstructions of the autonomic nervous system in the developing pelvis in six human embryos at between 5 and 8 weeks of development. Although one can object that six models cannot visualize all of the ENS in the developing embryo, we were able to provide a continuous account of the developmental appearance of relevant structures. A valid question is, nevertheless, whether all variation is accounted for. Although the answer is obviously 'no', differences between specimens could usually be explained as differences in degree of development rather than deviation from the expected morphology. The most important limitation of the present series is probably that the models still contain mistakes. Because the models were made in the software program Cinema4D, such mistakes can be corrected relatively easily.

In conclusion, the extrinsic innervation, both ganglionic cells and nerve fibres, in the lesser pelvis is organized in a similar fashion to that in its abdominal counterpart. Its three topographically separate preganglionic connections can be ascribed to differences in developmental timeline and its bilateral appearance to local peritoneal topography. Up to and including CS23 pelvic innervation is phenotypically indifferent with respect to sexual dimorphism.

## 5 DATA AVAILABILITY STATEMENT:

The data that support the findings of this study are available in the supplementary material of this article.

## CONFLICTS OF INTEREST

None declared.

## AUTHOR CONTRIBUTIONS

N.K. participated in data collection, analysis and visualization, and wrote the manuscript. J.H. and C.H. participated in data analysis and interpretation. G.M. and N.K. were responsible for formatting all Figures. S.E.K. participated in data analysis and interpretation, provided guidance, and edited the manuscript. W.H.L. conceived the study, provided guidance and assisted with data interpretation and preparation of the manuscript.

## Supporting information

Fig S1

Fig S2

Fig S3

Fig S4

Fig S5
