# Supplementary material for: Extrinsic innervation of the pelvic organs in the lesser pelvis of human embryos
Source: J Anat. 2020 Jun 27;237(4):672–88. doi: 10.1111/joa.13229 (PMC7495285; doi:10.1111/joa.13229)

Extrinsic innervation of the pelvic organs in the lesser pelvis of human embryos

Nutmethee Kruepunga[1,2], Jill P.J.M. Hikspoors[1], Cindy J.M. Hülsman[1], Greet M.C. Mommen[1],
S. Eleonore Köhler[1], Wouter H. Lamers[1*,3]

Supplemental Figure 4: Interactive 3D pdfs of the extrinsic innervation in the lesser pelvis from CS18 – CS22. Download the PDF-file (3D PDF can be opened on any computer as long as it contains Adobe PDF reader).

The 3D PDF becomes activated after 'clicking' with the mouse on the embryo. A toolbar appears at the top of the screen that includes the option 'model tree'. The model tree displays a material list of structures in the upper box and preset viewing options in the lower box. The list of visible structures can be modified by marking or unmarking a structure.

Furthermore, a structure can be rendered transparent by selecting that option from the dropdown menu after selecting the structure with the right mouse button. To manipulate the reconstruction, press the left mouse button to rotate it, the scroll button to zoom in or out, and the left and right mouse buttons simultaneously to move the embryo across the screen. The slicer button in the toolbar allows cross sections to be made. The plane of section can be adjusted with the offset and tilt options. To switch between reconstructions use the up or down arrows in the toolbar at the top.

The colour code is identical to that in the Figures and all structures are listed by name in the 'model tree'.

CS18 (44 days)

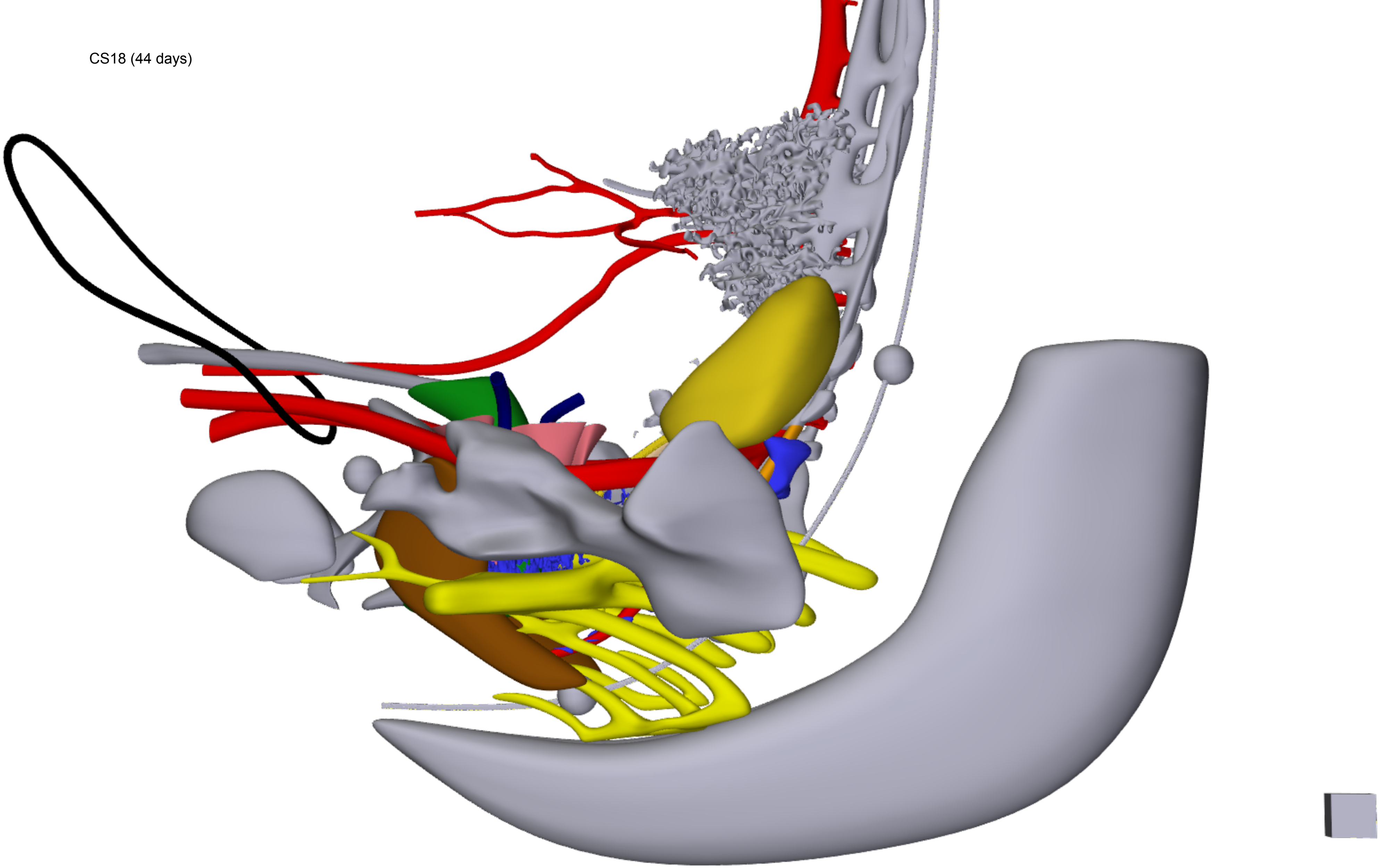

CS20 (49 days)

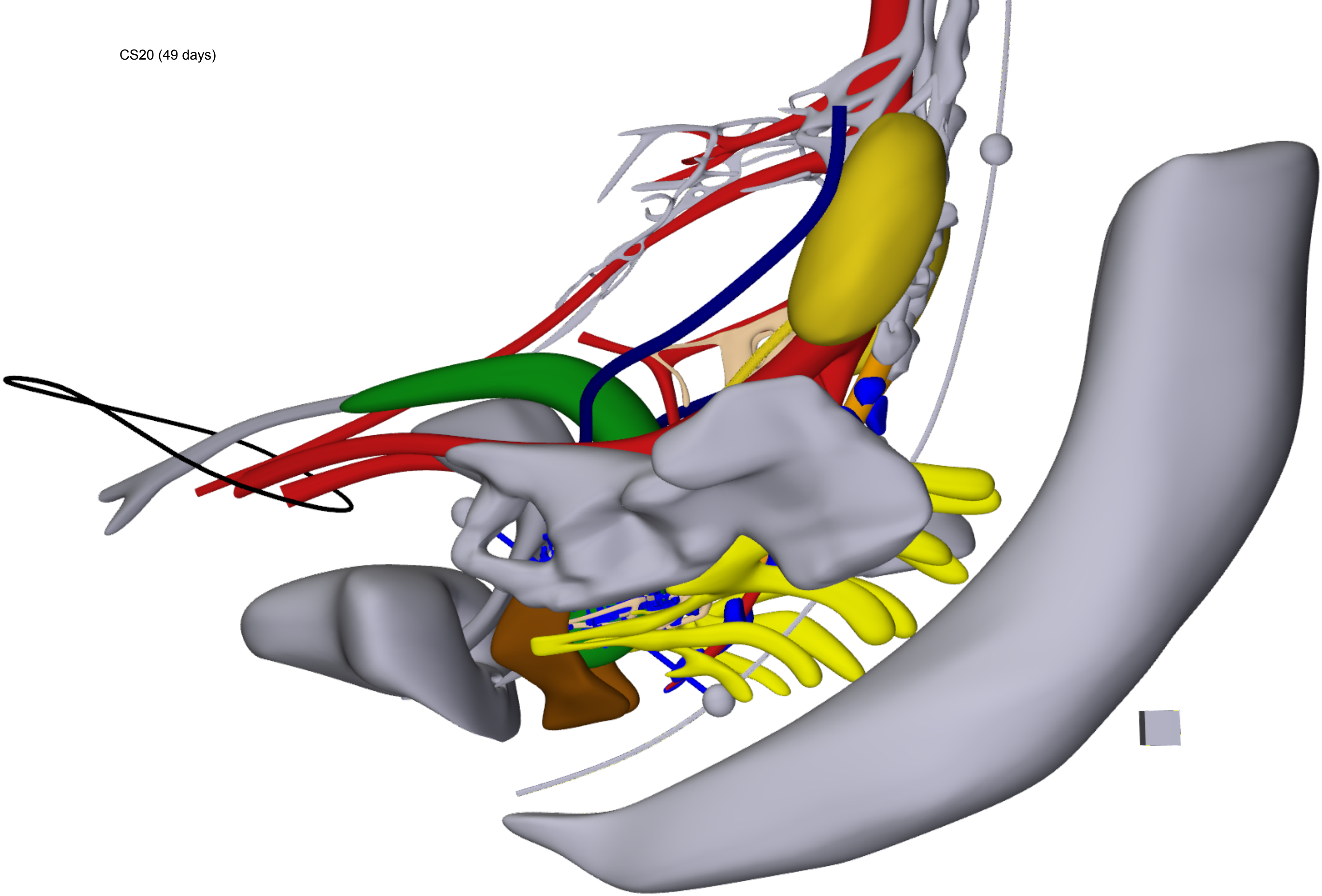

CS22 (5[illegible]

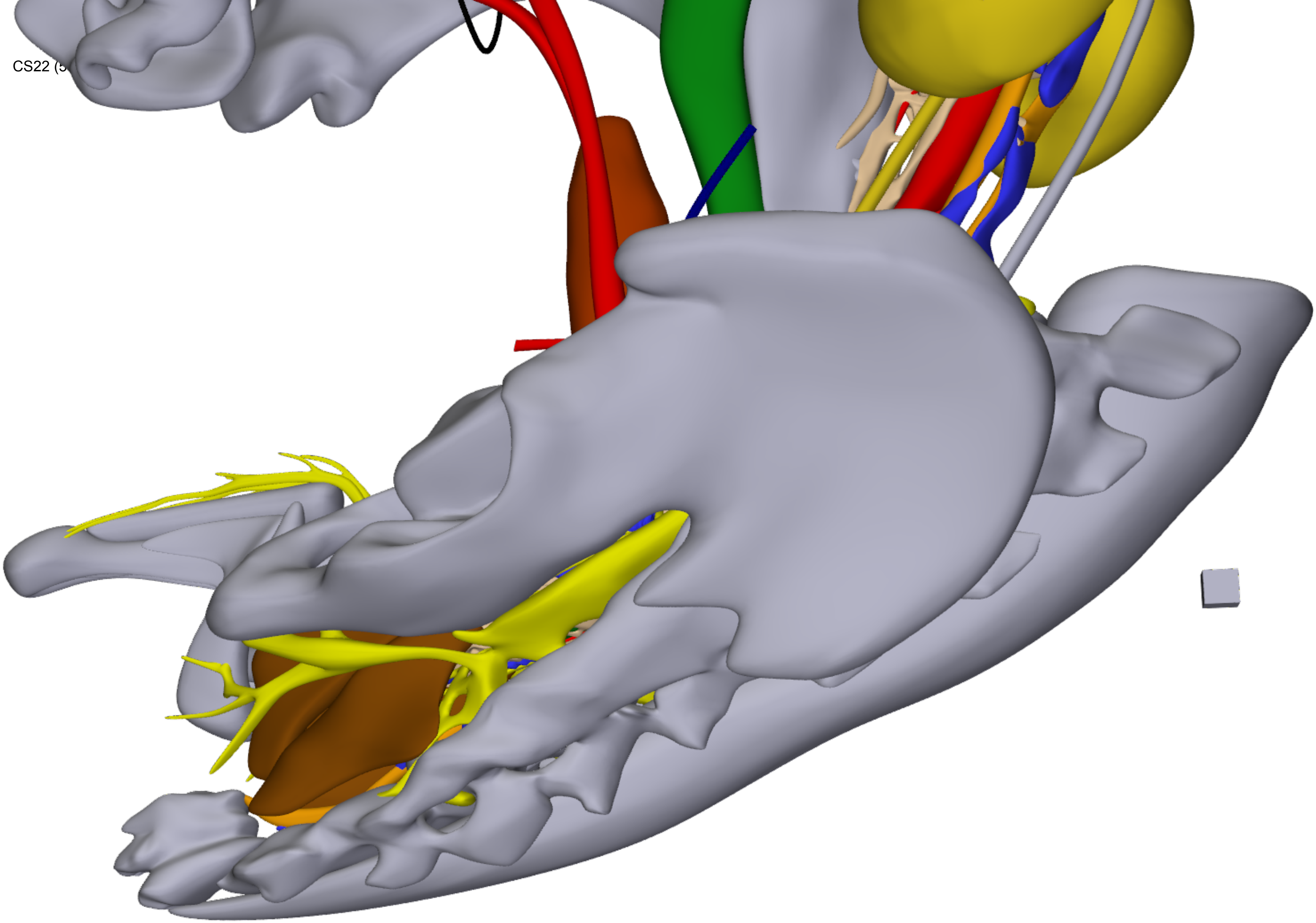

Supplement: Supplementary file 4 — Fig S4 [file JOA-237-672-s004.pdf]
